# Microbial perspective of inhibited carbon turnover in Tangel humus of the Northern Limestone Alps

**DOI:** 10.1111/1758-2229.13215

**Published:** 2023-12-07

**Authors:** Theresa Rzehak, Nadine Praeg, Harald Zink, Alois Simon, Clemens Geitner, Paul Illmer

**Affiliations:** ^1^ Department of Microbiology Universität Innsbruck Innsbruck Austria; ^2^ Department of Geography Universität Innsbruck Innsbruck Austria; ^3^ Department of Forest Planning Office of the Tyrolean Government Innsbruck Austria

## Abstract

Tangel humus primarily occurs in montane and subalpine zones of the calcareous Alps that exhibit low temperatures and high precipitation sums. This humus form is characterized by inhibited carbon turnover and accumulated organic matter, leading to the typical thick organic layers. However, the reason for this accumulation of organic matter is still unclear, and knowledge about the microbial community within Tangel humus is lacking. Therefore, we investigated the prokaryotic and fungal communities along with the physical and chemical properties within a depth gradient (0–10, 10–20, 20–30, 30–40, 40–50 cm) of a Tangel humus located in the Northern Limestone Alps. We hypothesized that humus properties and microbial activity, biomass, and diversity differ along the depth gradient and that microbial key players refer to certain humus depths. Our results give the first comprehensive information about microbiota within the Tangel humus and establish a microbial zonation of the humus. Microbial activity, biomass, as well as microbial alpha diversity significantly decreased with increasing depths. We identified microbial biomarkers for both, the top and the deepest depth, indicating different, microbial habitats. The microbial characterization together with the established nutrient deficiencies in the deeper depths might explain reduced C‐turnover and Tangel humus formation.

## Graphical abstract

Tangel, a humus form frequently found in Northern Alps, is characterized by thick organic layers due to limited carbon turnover. We proved poor nutrient conditions, especially towards deeper humus depths, and found a distinct microbial zonation and biomarkers along the humus profile. The study provides first insights regarding abundance and diversity of bacteria, archaea and fungi within this humus form.

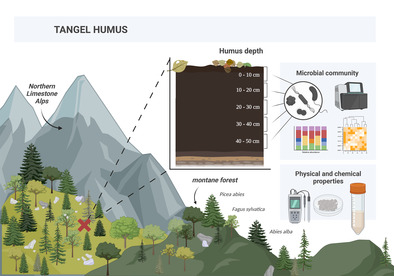

## INTRODUCTION

Tangel is one of the main terrestrial humus forms in the European humus classification system and primarily occurs at montane and subalpine zones of the Northern and Southern Limestone Alps with low mean annual temperatures and high annual precipitation sums (Kolb and Baier, 2001; Ponge et al., 2010). The vegetation at a Tangel site usually consists of mixed‐species or pure coniferous montane forests (*Fagus sylvatica* L., *Abies alba* Mill., *Picea abies* (L.) H. Karst.) and dwarf shrubs (*Pinus mugo*, shrubs of the Ericaceae family) (Kolb & Baier, 2001; Olleck et al., 2020). The Tangel humus is characterized by exceptionally thick organic layers (up to 100 cm thickness) (Kolb & Baier, 2001; Kolb & Kohlpaintner, 2018; Zanella, Ponge, Jabiol, et al., 2018), a high water storage capacity (Olleck et al., 2022), a steep pH gradient, ranging from lower pH values within the upper horizons to higher values within the organic layers contacting the bedrock (Kolb & Baier, 2001; Kolb & Kohlpaintner, 2018) and a slow biodegradation (Zanella, Ponge, Jabiol, et al., 2018). Podsols formed under cold and moist climate conditions exhibit thick organic layers similar to Tangel humus layers, but in contrast, Podsols exhibit an acidic pH within the entire humus layer (Grand & Lavkulich, 2011) and should not be confused with Tangel humus whose layers primarily emerge on base‐rich bedrock (mainly calcareous) and have a higher pH (Zanella, Ponge, & Briones, 2018).

The characteristic thick organic layers of a Tangel humus are a result of organic matter accumulation due to limited carbon turnover (Wang et al., 2019). In the case of the Tangel humus, it is supposed that slowly decomposing litter developed by the associated plant community (Kolb & Kohlpaintner, 2018) and negative, climatic impacts on the decomposing pedofauna (Ponge et al., 2010) could be reasons for the slow carbon turnover and the subsequent formation of these thick organic layers. Also, microorganisms could play an important role, as they are drivers of organic matter decomposition in humus (Wang et al., 2021), suggesting that a reduced decomposition activity impedes carbon turnover (Jastrow et al., 2007). The carbon within humus compounds is hard to metabolize by microorganisms (Martinez et al., 2013), which points to a supra‐regional issue microorganisms might have when considering the thick Tangel layers. The decomposition rate depends on the abundance, activity, and community composition of soil microorganisms (Bai et al., 2016; Cleveland et al., 2007). The mineralization of organic matter in soils is often coupled with nutrient availability (Kirkby et al., 2014), pH (Leifeld et al., 2013), and moisture (Yun et al., 2019), and strong impacts of these properties on the microbial community have been reported for alpine soils (Praeg et al., 2019). However, important microbial processes (e.g., organic matter decomposition, phosphate solubilization, nitrogen fixation) and microbial key players within the Tangel humus are largely unknown. In this context, we provide a DNA metabarcoding‐based description of the microbial communities in different depths of Tangel humus, along with physical, chemical, and microbial properties.

We hypothesize that (i) abiotic properties allow a characterization of different depths within the Tangel humus; (ii) microbial activity and biomass as well as community structure depend on depth; (iii) bacteria, archaea, and fungi are differentially altered with depth; (iv) microbial key players for Tangel humus and microbial biomarkers for different depths within the humus can be detected.

## EXPERIMENTAL PROCEDURES

### Humus profile description and sampling

We used the forest site classification (Forest Site Classification Tyrol, 2018) to select a representative area with homogeneous soil characteristics. The forest site under investigation is located in the Leutasch Valley within the Northern Limestone Alps, 20 km northwest of Innsbruck (Tyrol) in Austria (Figure A9). The area lies 1225 m above sea level in a northwest‐facing steep (25°), straight middle slope. As this place is difficult to access, the mixed‐species montane forest with *Fagus sylvatica*, *Abies alba*, and *Picea abies* is hardly managed. The mean annual temperature for the period from 1981 to 2010 was approximately 5.1°C, with an annual precipitation of 1400 mm (ZAMG et al., 2015). Precipitation is highest in the summer months, and the period with snow cover in winter and spring is long. The geological substrate for soil formation consists of slope debris from limestone pebbles of the Wetterstein formation, with a high carbonate content (>90%), mainly originating from a steep rock face a few 100 m up the slope. This rollover process can still be seen today in rock fragments on the surface. The average thickness of the total humus layer (50 cm, sometimes more) indicates that it must be classified as Pachytangel, according to Zanella et al. (2011). All characteristics fit the definition of a Tangel humus, which is distinguished from other humus forms by thick organic layers consisting of an OF and an OH horizon (together >10 cm), combined with a thinner C or A horizon (C/A < ½ OH) (Olleck et al., 2021; Zanella, Ponge, Jabiol, et al., 2018). Based on the WRB classification (IUSS Working Group WRB, 2014), the soil type can be designated as Folic Histosol. The content of mineral fragments was strongly increased in the deepest depth. Notably, mineral fragments were present in all depths, indicating a constant input of inorganic debris to the surface. Also, roots and macro‐residues (consisting of litter and wood pieces) appeared at all depths, but their amounts decreased towards the deeper depths (Table A1).

Considering the small‐scale structure of the forest type, we carefully selected a representative site for sampling. Samples were taken at three spots, each lying approximately 1 m apart from one another. At each spot, five different depth intervals (0–10, 10–20, 20–30, 30–40, 40–50 cm) were sampled, leading to a total of 15 single samples.

Of each sample, a proportion was directly used to investigate the coarse material fractions. Using a 2‐mm sieve, the coarse material was separated from the fine earth fraction and then manually divided into mineral fragments, roots, and macro‐residues (pieces of wood, and needles, among others). The dry weight (DW [g g^−1^ soil]) of the coarse material fractions was determined by drying them at 105°C for 24 h. Immediately after sampling, the humus samples were transported to the laboratory and frozen at −20°C for all chemical and microbiological analyses.

### Physical and chemical properties

After defrosting the humus samples, they were sieved to 2 mm, and the dry weight (DW [g g^−1^ soil]) was determined by drying them at 105°C for 24 h. The oven‐dried humus was then ignited in a muffle furnace for 4 h at 430°C to estimate the organic matter content (OM [g g^−1^ DW]) (Schinner, 1996), and subsequently, the samples were analysed on a CN analyser (Truspec CHN Macro, Leco, MI, USA) to determine the total carbon (TC [mg C g^−1^ DW]) and nitrogen (TN [mg N g^−1^ DW]) contents. Electrical conductivity (EC [μS cm^−1^]) was measured in deionized water, and soil pH was determined in a CaCl_2_ solution (0.01 M) at a 1:2.5 (w/v) soil: water ratio. Plant‐available phosphorus (P [μg P g^−1^ DW]) was measured in LiCl solution, applying the Olsen method as described in Schinner (1996). Total dissolved carbon (TDC [mg C g^−1^ DW]), nitrogen (TDN [mg N g^−1^ DW]), and dissolved organic carbon (DOC [mg C g^−1^ DW]) were extracted by mixing 5 g of fresh soil with 45 mL of distilled water, and the contents were measured using a TOC‐L analyser (Shimadzu, Japan).

### Microbial activity and biomass

Upper humus depths contain a high amount of macro‐residues (Table A1) of which the dominant compound usually is cellulose (Xu et al., 2021). Furthermore, cellulose is the most produced organic compound on Earth (McNamara et al., 2015). So, we investigated the activity of cellulolytic microorganisms by measuring the cellulase activity (CA [μg glucose equivalent g^−1^ DW d^−1^]) in our samples. Therefore, samples were incubated with CM‐cellulose (substrate) for 24 h at 50°C, and released sugars were measured colorimetrically following the protocol described in Schinner (1996). Basic soil respiration and substrate‐induced respiration (SIR) (for the calculation of microbial biomass) were determined on an EGA61‐Soil respiration Device (ADC BioScientific, UK). Samples were filled into acrylic glass tubes, closed with polystyrene foam pads, and aerated with a continuous stream of ambient air (humidified and tempered to 22°C). The CO_2_ released from the samples was recorded for 6 h with an infrared gas analyser (IRGA) to calculate the basic soil respiration (BR [μg CO2 g^−1^ DW h^−1^]). Afterwards, glucose (1%) was added to the samples, and the CO_2_ release was further recorded for 12 h (substrate‐induced respiration method). The maximum CO_2_ release was used to calculate the microbial biomass (Cmic [μg C g^−1^ DW]) according to Anderson and Domsch (1978). The metabolic quotient (MQ) was calculated as the quotient of basic soil respiration (BR) and microbial biomass (Cmic).

### Microbial diversity

#### DNA extraction and amplicon sequencing

Genomic DNA was extracted from 250 mg of fresh material using the NucleoSpin®Soil Kit (Macherey‐Nagel, Germany) with SL1 lysis buffer and 50 μL enhancer. The DNA quality and quantity were checked via UV/VIS spectrophotometry on a NanoDrop 2000cTM (Thermo Fisher Scientific Inc, Germany) and QuantiFluor® dsDNA Dye (Promega, Germany). DNA extracts were submitted to Microsynth Austria GmbH where amplicon sequencing on an Illumina MiSeq platform (v2) performing a paired‐end run (250 bp) was carried out. Sequencing was performed targeting the V4 region of the 16S rRNA gene (primer pair 515f/806r, Caporaso et al., 2011) for prokaryotes and the ITS2 region (primer pair ITS3/ITS4, White et al., 1990) for fungi.

#### Sequence data processing

After trimming barcodes and primers, sequence reads were processed and analysed using mothur v.1.44.1 (Schloss et al., 2009). Paired‐end reads with a phred score ≥25 for 16S rRNA and ≥20 for ITS2 were merged. Sequences not fulfilling certain quality criteria (no appropriate length, >8 ambiguous bases, homopolymers) were discarded. The 16S rRNA sequences were aligned against the SILVA SSU database (release 138) (Quast et al., 2012) using kmer searching. Potentially erroneous sequences (due to sequencing errors) were removed by pre‐clustering the sequences (Huse et al., 2010). Chimeric sequences were identified using VSEARCH (Rognes et al., 2016) and discarded. Sequences were classified using the SILVA SSU database (release 138, December 2019) (Quast et al., 2012) for 16S rRNA sequences and the UNITE database (v. 8.2 with dynamic use of clustering thresholds) (Abarenkov et al., 2020) for ITS2 sequences. For classification, the Wang approach was applied (Wang et al., 2007), which assigns query sequences to the taxonomy with the highest probability of containing specific kmers (bootstrap cutoff value 80%). Sequences assigned to unwanted lineages were removed from 16S rRNA and ITS2 libraries. The algorithm OptiClust (Westcott et al., 2017) was used to assign sequences to operational taxonomic units (OTUs, 97% identity). Rare OTUs with less than five observations within the dataset were discarded.

#### Droplet‐digital PCR

For the quantification of total archaeal, bacterial, and fungal abundances, droplet‐digital PCR (ddPCR) was conducted on a QX200TM droplet digital PCR system, using a QX200TM droplet generator (Bio‐Rad, Munich, Germany). The archaeal and bacterial 16S rRNA genes were targeted using the primer pairs 787f/1059r (Yu et al., 2005) and 338F/518r (Ghyselinck et al., 2013; Orschler et al., 2019), respectively, as described in Praeg et al. (2021). The fungal 18S rRNA gene was quantified using the primer pair FungiQuant‐F/FungiQuant‐R (Liu et al., 2012; Unterwurzacher et al., 2018), as described in Gorfer et al. (2022). The amplification conditions for archaea were as follows: initial denaturation step (5 min, 95°C), 45 cycles of denaturation (30 s, 95°C), annealing (30 s, 57.5°C), and elongation (1 min, 72°C), with two final steps for droplet stabilization (5 min, 4°C and 5 min, 95°C). The amplification conditions for bacteria and fungi were the same as those for archaea, albeit with the following variations: 40 cycles of denaturation, annealing (58°C), elongation for bacteria; 40 cycles of denaturation, annealing (1 min, 52.5°C), elongation (2 min) for fungi.

### Data analysis

#### Physical, chemical and microbial properties

All statistical analyses were conducted in R v.4.0.3 (R Core Team, 2020), using the packages *stats*, *ggpubr*, *rstatix*, and *ggplot2*. The normal distribution of the data was tested using the Shapiro–Wilk test. Differences among the physical and chemical parameters, microbial abundance, and activity in relation to the five depth levels of the Tangel humus were tested with one‐way analysis of variance (ANOVA) and post‐hoc analysis (Fisher's LSD post‐hoc test) at a significance level of *p* < 0.05. Soil respiration, microbial biomass, and cellulase activity data were log‐transformed for data normalization prior to statistical analysis.

#### Microbial community data

The alpha diversity was estimated in R (R Core Team, 2020) using the *iNEXT* package (Chao et al., 2014; Hsieh et al., 2016). Alpha diversity estimation in different humus depths was done by calculating the effective number of species (based on inverting a diversity index, resulting in the number of species that must be present to receive the calculated index value) with different sensitivities to common and rare species for any sample size (read numbers) up to the double sample size. The *iNEXT* function was used to perform fair comparisons of different measures of Hill numbers (the effective number of species) among samples with different sample sizes (without read count normalization, therefore losing no diversity data). We used three measures of Hill numbers: species richness, Shannon diversity (= exponential Shannon index), and Simpson diversity (= inverse Simpson index). The three species diversity measures have a different sensitivity to the relative abundance of species: Species richness gives equal weight to common and rare species; Shannon diversity gives less weight to rare species; Simpson diversity favours abundant species. Diversity estimates were calculated for rarefied (interpolated) and extrapolated (predicted) sample means (*n* = 3 per humus depth) up to a certain sample size and plotted via sample‐size‐based rarefaction/extrapolation (R/E) curves.

To compare the community structure among the samples, their sequencing depths (number of reads) were normalized. We tested the applicability of four different normalization methods (see respective section in the supplement and Figure A1A,B) and decided to use rarefaction for read count normalization. Data were rarefied to even depth using the *phyloseq* package (McMurdie & Holmes, 2013). The total 16S rRNA read numbers ranged from 30,269 to 71,746 reads per sample and were rarefied to sample sums of 30,238 reads. The total ITS2 read numbers ranged from 21,539 to 66,612 reads per sample and were rarefied to sample sums of 21,517 reads. The 16S rRNA libraries contained 4330 OTUs and the ITS2 libraries 1391 OTUs.

Differences among the microbial communities from the five Tangel humus depths were established by using a one‐way PERMANOVA (based on Bray–Curtis dissimilarities) and by applying the *adonis* function included in the *vegan* package (Oksanen et al., 2019). To test if dissimilarities within depths are similar, the analysis of similarities (ANOSIM) using the *anosim* function in the *vegan* package (Oksanen et al., 2019) was applied. Prokaryotic and fungal beta diversity was visualized by performing a PCoA based on Bray–Curtis dissimilarities. Relative abundances were calculated at phylum, class, and family level for each humus depth by combining three samples per depth to a mean rank. Relative abundances in all five humus depths were visualized for the most abundant prokaryotic and fungal classes (*n* = 10) or families (*n* = 50).

The following beta‐diversity and biomarker analyses were conducted using the *microeco* package (Liu et al., 2021). Redundancy analysis (RDA) was performed with default settings to investigate which environmental variables can explain the variation of the microbial communities in different Tangel depths. Significant correlations (Pearson) between environmental variables and the Bray–Curtis distance matrix were determined by a Mantel test. The LEfSe tool (Segata et al., 2011) was employed to identify biomarker taxa for a certain humus depth, including the calculation of effect sizes. The random forest approach was applied to determine taxa exhibiting significantly different relative abundances among the five humus depths. The resulting taxa were ranked according to the mean decrease accuracy, which is a measure of the importance of the features (An et al., 2019). Pearson's correlation analysis was performed to analyse if and how relative abundances of biomarkers were connected to environmental variables. Correlations were visualized via a correlation heatmap.

## RESULTS

### Physical and chemical properties

The DW ranged from 0.32 g g^−1^ soil in the top depth (0–10 cm) to 0.37 g g^−1^ soil in the deepest depth (40–50 cm) and differed significantly between depths 0–10 cm, 10–20 and depths 30–40 cm, 40–50 cm (Table 1; Figure A2 A). pH values increased gradually from 5.22 within the top depth (0–10 cm) to 6.74 within the deepest depth (40–50 cm), which differed significantly from the uppermost depths (0–10 cm, 10–20 cm) (Table 1; Figure A2B). The EC was significantly higher within the top depth at 0–10 cm depth (126 μS cm^−1^) compared with the deeper humus depths (20–30 to 40–50 cm, EC of 89.2 to 80.9 μS cm^−1^) (Table 1; Figure A2C). The contents of OM, TC, and TN significantly decreased with increasing sampling depth (from 0–10 to 40–50 cm) (Table 1; Figure A2D–F), whereas the DOC did not change significantly (Table 1; Figure A2J) from the top (0–10 cm) to the bottom (40–50 cm) of the Tangel humus. The plant‐available P and TDN contents were highest within the top depth (0–10 cm) (46.63 μg P g^−1^ DW and 0.46 mg TDN g^−1^ DW) and significantly declined towards the deeper humus depths (10–20 to 40–50 cm), reaching a low P content of 1.26 μg P g^−1^ DW and 0.17 mg TDN g^−1^ DW in the lowest depth (40–50 cm) (Table 1; Figure A2G,H). The TDC and DOC contents were lowest at a depth of 10–20 cm (0.63 mg TDC g^−1^ DW, 0.54 mg DOC g^−1^ DW) and slightly, though not significantly, increased with increasing depth (from 10–20 to 40–50 cm) (Table 1; Figure A2I,J). Thus, the ratios of dissolved carbon fractions (DOC and TDC) to total carbon (TC) increased significantly in the deeper humus depths (from 20–30 to 40–50 cm) (Table 1). The total C/N ratio varied between 22.6 and 21.5 and did not respond significantly to humus depth (Table 1; Figure A2K). The TDC/TDN ratio increased significantly from 1.8 within the top depth (0–10 cm) to 4.1 within the deepest depth (40–50 cm) (Table 1; Figure A2L).

**TABLE 1:** Physical and chemical properties, microbial activity and microbial abundance data for five humus depth steps given as means (*n* = 3) and respective standard deviations (italics).

Depth [cm]	OM [g g^−1^ DW]	DW [g g^−1^ soil]	pH	EC [μS cm^−1^]	TC [mg g^−1^ DW]	TN [mg g^−1^ DW]	C/N ratio	TDC [mg g^−1^ DW]	DOC [mg g^−1^ DW]	TDN [mg g^−1^ DW]	TDC/TDN ratio	P [μg g^−1^ DW]	CA [μg glucose g^−1^ DW d^−1^]
0–10	0.88^a^	0.32^a^	5.22^a^	125.98^a^	473.50^a^	21.11^a^	22.46^a^	0.84^a^	0.64^a^	0.46^a^	1.82^a^	46.63^a^	1809.81^a^
	*0.06*	*0.02*	*0.69*	*7.34*	*20.54*	*1.42*	*0.85*	*0.27*	*0.19*	*0.12*	*0.15*	*18.55*	*382.01*
10–20	0.80^ab^	0.32^a^	5.48^a^	89.15^ab^	433.83^ab^	19.20^ab^	22.61^a^	0.63^a^	0.51^a^	0.28^b^	2.35^ab^	17.37^b^	1292.96^ab^
	*0.06*	*0.00*	*0.86*	*25.34*	*17.93*	*0.94*	*0.45*	*0.16*	*0.08*	*0.04*	*1.00*	*4.99*	*284.74*
20–30	0.71^bc^	0.34^ab^	5.67^ab^	85.62^b^	385.67^bc^	17.56^bc^	21.91^a^	0.66^a^	0.56^a^	0.24^b^	2.77^abc^	11.40^b^	876.45^bc^
	*0.10*	*0.03*	*0.87*	*31.32*	*51.08*	*0.98*	*1.66*	*0.13*	*0.08*	*0.05*	*0.73*	*11.35*	*586.90*
30–40	0.63^cd^	0.36^b^	6.37^ab^	80.43^b^	338.67^cd^	15.74^c^	21.54^a^	0.67^a^	0.61^a^	0.19^b^	3.72^cd^	2.29^b^	580.17^c^
	*0.03*	*0.01*	*0.20*	*21.18*	*29.24*	*1.42*	*0.68*	*0.07*	*0.09*	*0.03*	*1.07*	*1.08*	*171.75*
40–50	0.56^d^	0.37^b^	6.74^b^	80.90^b^	300.17^d^	13.39^c^	22.49^a^	0.69^a^	0.64^a^	0.17^b^	4.05^d^	1.26^b^	547.03^c^
	*0.03*	*0.02*	*0.11*	*3.03*	*5.62*	*0.88*	*1.39*	*0.04*	*0.06*	*0.03*	*0.53*	*0.74*	*51.77*

BR [μg CO2 g^−1^ DW h^−1^]	Cmic [μg C g^−1^ DW]	Bacteria [copies g^−1^ DW]	Bacteria log 10	Archaea [copies g^−1^ DW]	Archaea log 10	Fungi [copies g^−1^ DW]	Fungi log 10	BR/TC ratio	MQ ratio	Archaea/fungi ratio	Bacteria/archaea ratio	Bacteria/fungi ratio
34.97^a^	6436.98^a^	1.39E+10	10.14^a^	4.43E+07	7.65^a^	4.13E+08	8.56^a^	73.51^a^	0.40^a^	0.89^a^	1.33^a^	1.19^a^
*7.44*	*1431.01*	*1.98E+09*	*0.06*	*2.97E+06*	*0.03*	*2.16E+08*	*0.29*	*12.82*	*0.02*	*0.03*	*0.01*	*0.03*
15.10^b^	3377.27^b^	1.46E+10	10.16^a^	5.21E+07	7.71^ab^	3.04E+08	8.41^ab^	34.61^b^	0.33^ab^	0.92^ab^	1.32^a^	1.21^ab^
*5.66*	*937.46*	*1.82E+09*	*0.06*	*1.03E+07*	*0.09*	*2.23E+08*	*0.30*	*12.19*	*0.05*	*0.04*	*0.02*	*0.04*
8.49^bc^	1905.27^bc^	1.16E+10	10.04^a^	4.96E+07	7.67^a^	2.23E+08	8.24^abc^	22.44^bc^	0.27^bc^	0.93^ab^	1.31^a^	1.22^ab^
*4.15*	*1023.17*	*4.64E+09*	*0.19*	*2.05E+07*	*0.18*	*2.01E+08*	*0.36*	*12.55*	*0.08*	*0.05*	*0.02*	*0.04*
5.20^c^	1114.23^c^	1.01E+10	10.00^a^	6.19E+07	7.76^ab^	1.13E+08	8.01^bc^	15.13^cd^	0.23^c^	0.97^bc^	1.29^ab^	1.25^bc^
*1.93*	*421.68*	*5.93E+08*	*0.03*	*2.88E+07*	*0.21*	*6.64E+07*	*0.24*	*4.24*	*0.04*	*0.06*	*0.04*	*0.04*
1.30^c^	586.97^c^	1.10E+10	10.04^a^	8.86E+07	7.95^b^	6.03E+07	7.77^c^	4.32^d^	0.04^d^	1.02^c^	1.26^b^	1.29^c^
*0.48*	*165.80*	*1.87E+09*	*0.07*	*9.22E+06*	*0.05*	*1.55E+07*	*0.11*	*1.58*	*0.06*	*0.02*	*0.01*	*0.02*

*Note*: Significant differences among humus depth [cm] are indicated by different superscript letters.

Abbreviations: archaea, archaeal gene abundance; archaea/fungi, ratio of archaeal gene abundance to fungal gene abundance; bacteria, bacterial gene abundance; bacteria/archaea, ratio of bacterial gene abundance to archaeal gene abundance; bacteria/fungi, ratio of bacterial gene abundance to fungal gene abundance; BR, basal respiration; BR/TC, basal respiration to total carbon ratio; C/N, carbon to nitrogen content ratio; CA, cellulase activity; Cmic, microbial biomass; DOC, dissolved organic carbon; DW, dry weight; EC, electrical conductivity; fungi, fungal gene abundance; MQ, metabolic quotient; OM, organic matter; P, phosphorus content; TC, total carbon content; TDC, total dissolved carbon; TDC/TDN, total dissolved carbon to total dissolved nitrogen ratio; TDN, total dissolved nitrogen; TN, total nitrogen content.

### Effects of physical and chemical properties on microbial communities

Redundancy analysis revealed that the physical and chemical properties of the Tangel humus depths discriminated into two directions, thereby clustering the samples according to the different humus depths on both axes (Figure A8A,B). A subset of the soil properties that could explain most of the variation was selected by forward selection, including sampling depth as a co‐variate to eliminate autocorrelated variables for further analyses. The chosen variables that most represented the variations in prokaryotic and fungal RDA were OM, pH, TDC, and TDN (ordered by decreasing contribution to the variation of the model results). The significant influence of selected variables on the prokaryotic and fungal community structure was demonstrated by a Mantel test, resulting in Pearson's correlations shown in Table A5. The variables OM (Mantel *p* < 0.001), pH (*p* < 0.001), TDC (Mantel *p* < 0.01) and TDN (Mantel *p* < 0.01) were positively correlated with prokaryotic and fungal community composition.

**FIGURE 1:**
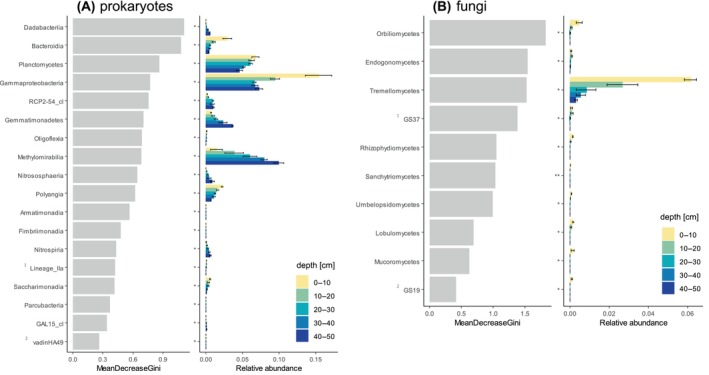
Bar plots representing differentially abundant classes identified by random forest analysis and ranked according to their importance (mean decrease accuracy as a measure of feature importance, left panel) and relative abundances among different humus depths [cm] (right panel). Asterisks represent the significances (**p < 0.01; *p < 0.05). (A) Prokaryotes (^1^Phylum Elusimicrobiota, ^2^Phylum Planctomycetota); (B) Fungi (^1^Phylum Ascomycota, ^2^Phylum Kickxellomycota).

**FIGURE 2:**
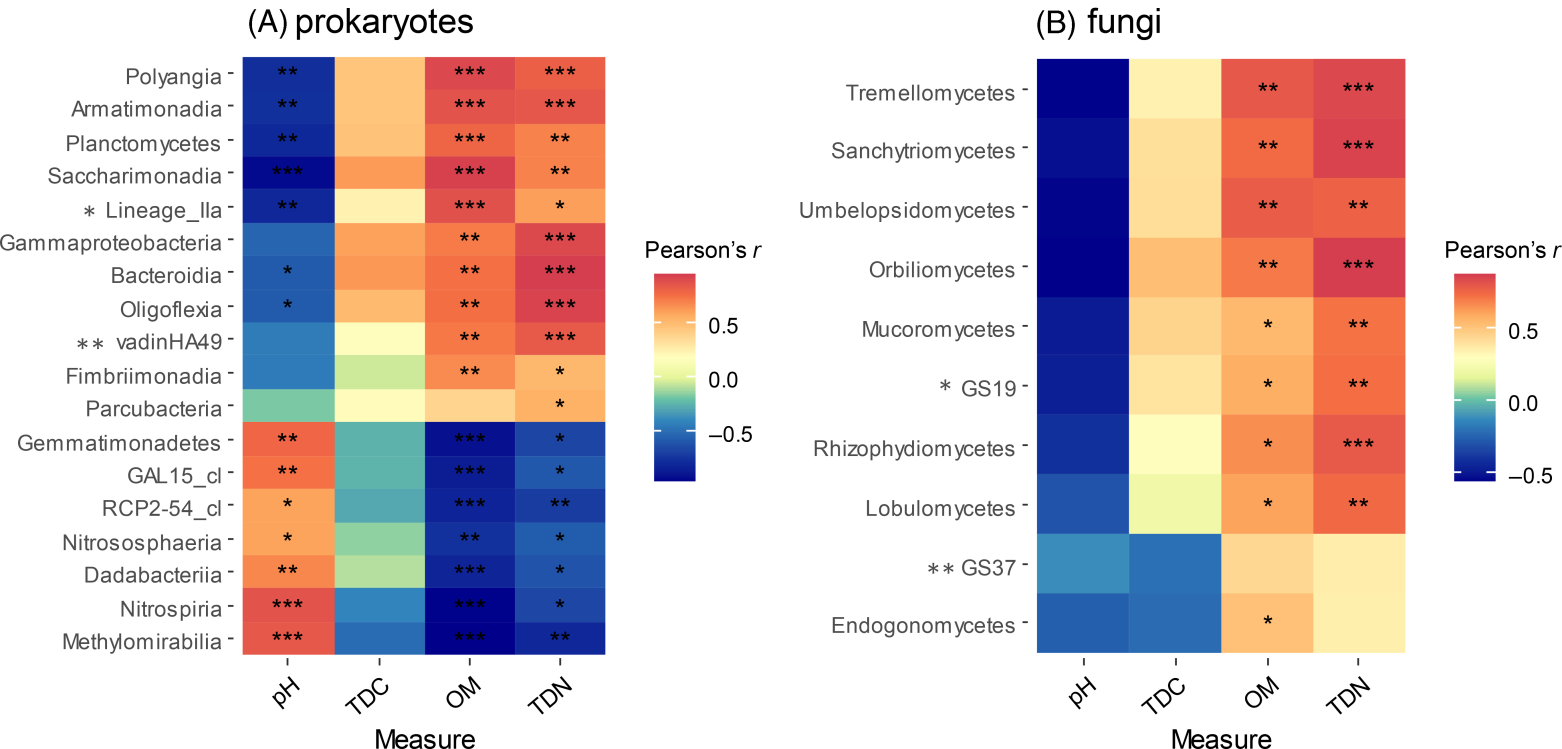
Heatmaps representing Pearson's correlations of differentially abundant classes (identified by random forest analysis) and selected chemical properties. (A) Prokaryotes (*Phylum Elusimicrobiota, **Phylum Planctomycetota); (B) Fungi (*Phylum Kickxellomycota, **Phylum Ascomycota).

To test whether OM, pH, TDC, and TDN caused significant changes in the abundances of prokaryotic and fungal classes (selected by random forest analysis, Figure 1) among different humus depths, correlation analysis was performed, and the results were plotted (Figure 2). The resulting heatmap showed that the selected prokaryotic classes were divided into two groups according to their correlation with chemical properties (Figure 2A). The first group (comprising 11 prokaryotic classes) was significantly negatively affected by pH but positively influenced by OM and TDN, whereas the second group (comprising 7 classes) was significantly positively affected by the pH and negatively influenced by OM and TDN. No significant correlation was found between the most important classes and TDC, but when the OM content had a significant positive or negative effect on the relative abundance, TDC performed similarly (Figure 2A). The selected fungal classes all showed a similar correlation pattern (Figure 2B), as the relative abundances of all classes were negatively correlated with pH and positively correlated with OM and TDN. Positive correlations with OM and TDN were mostly significant (except with the ascomycetal class GS37 and TDN with Endogonomycetes), whereas no taxon was significantly correlated with pH. According to their significant correlation with TDC, fungal classes were clustered into two groups: GS37 and Endogonomycetes were negatively correlated with TDC, whereas all other classes were slightly positively correlated with TDC (Figure 2B).

### Microbial activity and abundance

Cellulase activity (CA), basal respiration (BR), and microbial biomass (Cmic) exhibited the highest values in the upper humus depth (0–10 cm) and values decreased significantly with increasing humus depth (from 0–10 to 40–50 cm) (Table 1; Figure A3A–C).

Archaeal, bacterial, and fungal abundances were investigated in all Tangel humus depths (Figure A4 A–C). Bacterial gene abundances ranged from 1.0 × 10^10^ to 1.5 × 10^10^ gene copies g^−1^ DW, archaeal gene abundances from 4.4 × 10^7^ to 8.9 × 10^7^ gene copies g^−1^ DW, and fungal gene abundances from 6.0 × 10^7^ to 4.1 × 10^8^ gene copies g^−1^ DW (Table 1; Figure A4). The abundance of archaea significantly increased with increasing sampling depth (from 0–10 to 40–50 cm) (Table 1; Figure A4A), whereas that of bacteria decreased only slightly (not significantly) from the upper (0–10 cm) to the lower humus depth (40–50 cm) (Table 1; Figure A4B). Fungal abundance decreased significantly from the top (0–10 cm) to the lowest Tangel humus depth (40–50 cm) (Table 1; Figure A4C). These abundances resulted in a significant increase in the ratio of bacteria/fungi and archaea/fungi and a significant decrease in the ratio of bacteria/archaea with humus depth (0–10 to 40–50 cm) (Table 1; Figure A4D).

### Microbial community structure and diversity

Taxonomic identification at the class level of the prokaryotic and fungal community composition within sampled Tangel humus (all humus depths combined) showed that Alphaproteobacteria (22.17%) dominated the prokaryotic community, followed by Gammaproteobacteria (9.06%) (both classes are assigned to the phylum Proteobacteria) (Table A2). The fungal community within the Tangel humus (all humus depths combined) was dominated by Agaricomycetes (18.93%), Sordariomycetes (18.07%) and Leotiomycetes (17.01%) (Table A2). Considering the relative abundances of the 10 most abundant prokaryotic and fungal classes in the different depths revealed differences due to humus depth (Figure A5; Table A3). The relative abundances of Methylomirabilia*, Vicinamibacteria, Blastocatellia, and Thermoleophilia increased in the deeper depths, whereas those of Gammaproteobacteria*, Actinobacteria, Planctomycetes, Acidobacteriae, and Verrucomicrobiae decreased (* = significantly, Figure A5A; Table A3). Agaricomycetes constituted the most abundant fungal class in the upper depths, but their relative abundance decreased with increasing sampling depth (Figure A5B; Table A3). Similarly, the relative abundances of Tremellomycetes* and Eurotiomycetes decreased with increasing sampling depth (* = significant difference among depth gradient, Figure A5B; Table A3). The relative abundances at a lower taxonomic level (family) also showed differences due to humus depth (Figure A6A,B). A total of 18 prokaryotic and 10 fungal classes were found to be significantly different in terms of relative abundances across the five humus depths (Figure 1; Table A7). Dadabacteriia, Bacteroidia and Planctomycetes emerged as prokaryotic classes with the most distinct differences in relative abundance among the humus depths (Figure 1A), whereas Orbiliomycetes, Endogonomycetes and Tremellomycetes were the most important fungal classes (Figure 1B). Considering the most abundant classes with significant differences among the sampled humus depths, the relative abundances of Methylomirabilota and Gemmatimonadetes were significantly increased in the deeper depths, whereas those of Gammaproteobacteria, Planctomycetes and Polyangia were significantly decreased in the deeper humus depths (Figure 1A; Table A7). Tremellomycetes represented the most abundant fungal class, with significant differences among the different depths; their abundances were significantly higher in the upper compared to the deeper humus depths (Figure 1B; Table A7).

**FIGURE 3:**
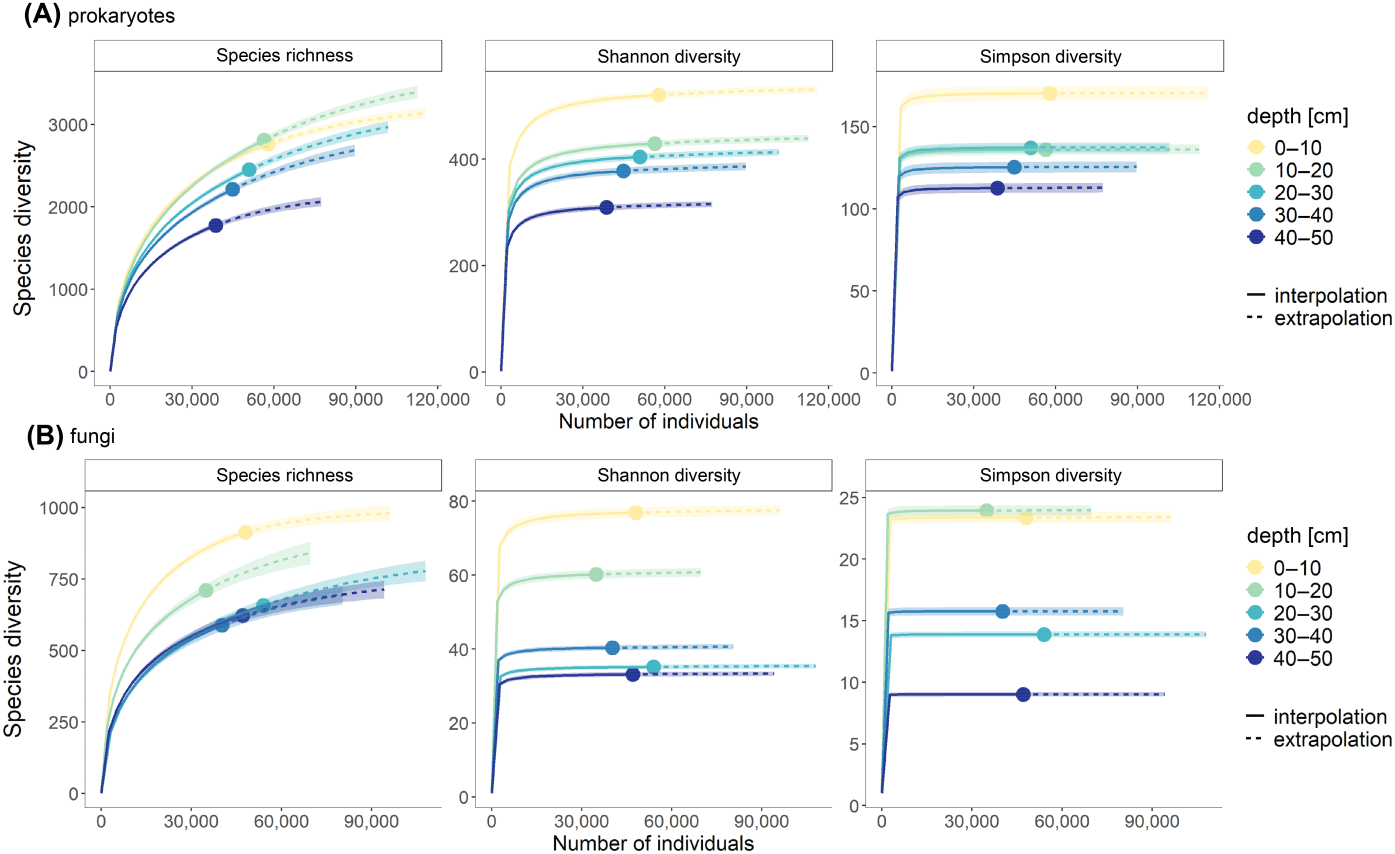
Sample‐size‐based rarefaction (solid line segment) and extrapolation (dotted line segment) sampling curves with 95% confidence intervals (shaded areas; based on a bootstrap method with 200 replications) of five humus depths [cm] (*n* = 3) separately by measures of Hill numbers: Species richness (left panel), Shannon diversity (= exponential of Shannon index, middle panel) and Simpson diversity (= inverse of Simpson index, right panel). The number of individuals refers to the sequencing depth (number of reads). (A) Prokaryotes; (B) Fungi.

Prokaryotic and fungal observed alpha diversity differed significantly among humus depths, except from prokaryotic Simpson and fungal Shannon values (no significant difference) (Figure 3 (dots) and Table A4). All alpha diversity measures of prokaryotes and fungi exhibited the highest values in the top depths (0–10 and 10–20 cm). Alpha diversity estimates, computed for interpolated and extrapolated sequencing depth revealed concordant results: a significant decline in the alpha diversity in prokaryotic and fungal communities was observed from the top depth (0–10 cm) to the deepest depth (40–50 cm) (Figure 3A,B). Prokaryotic species richness was similar between the two upper humus depths (0–10, and 10–20 cm depth), but when the sequencing depth was increased by extrapolation, the second depth (10–20 cm) showed a significantly higher species richness than the top humus depth (0–10 cm), as the rarefaction curves and 95% confidence intervals of both depths completely overlapped (Figure 3A, species richness). In the deeper depths, species richness was significantly lower than in the upper depths for any sequencing depth, as the 95% confidence intervals of these curves did not overlap (Figure 3A, species richness). Fungal species richness was lower compared with prokaryotic richness (Figure 3A,B); thus, fungal richness also decreased significantly in the deeper depths (Figure 3B). The uppermost humus depth exhibited a significantly higher fungal richness than the second depth (10–20 cm), and the three deeper depths (20–30, 30–40, 40–50 cm) showed concordant richness values at each sequencing depth (Figure 3B). Prokaryotic and fungal Shannon diversity was significantly decreased in the deeper depths, irrespective of the sequencing depth (Figure 3A,B; Shannon diversity), as the 95% confidence intervals of these curves did not overlap. Prokaryotic Simpson diversity was significantly lower in the deeper humus layer (0–10 cm compared with 40–50 cm), whereas the layers at 10–20 and 20–30 cm showed similar Simpson diversities (Figure 3A). The highest fungal Simpson diversity was observed in the upper humus depths (0–10 cm and 10–20 cm) (Figure 3B) and diversity significantly decreased in the deeper depths (40–50 cm) (Figure 3B).

**FIGURE 4:**
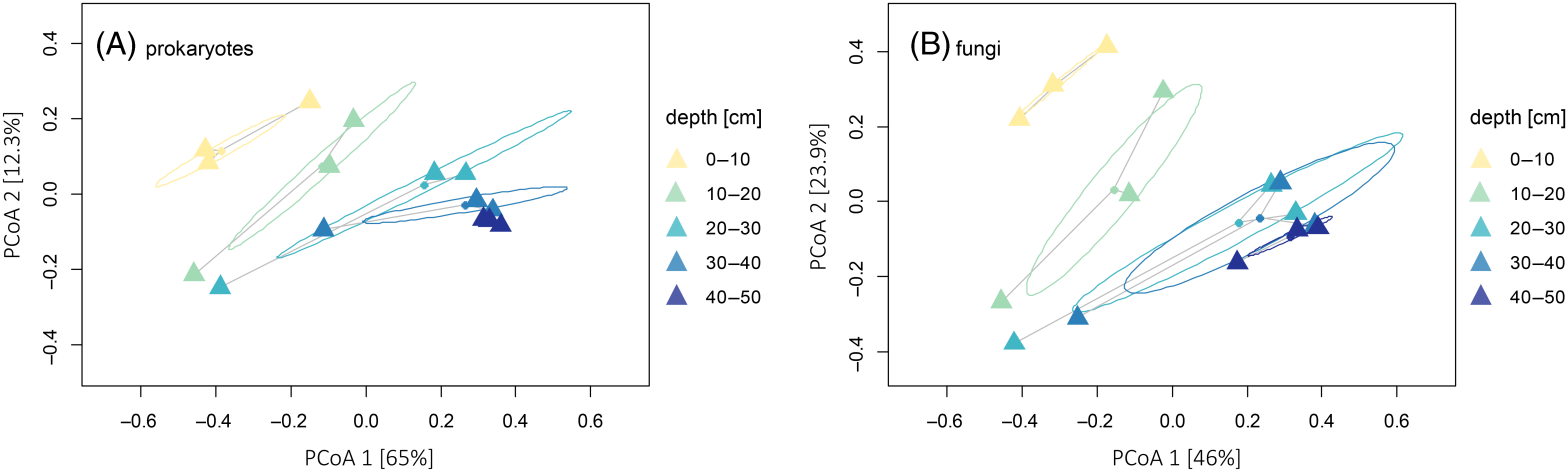
Heatmaps representing Pearson's correlations of differentially abundant classes (identified by random forest analysis) and selected chemical properties. (A) Prokaryotes (*Phylum Elusimicrobiota, **Phylum Planctomycetota); (B) Fungi (*Phylum Kickxellomycota, **Phylum Ascomycota).

The PERMANOVA analysis identified significant differences in the prokaryotic (*p* < 0.05, *R* ^2^ = 0.52) and fungal (*p* < 0.05, *R* ^2^ = 0.43) community compositions among the different humus depths. The microbial communities significantly differed among the five depths, with significant differences in group similarities (prokaryotes: ANOSIM *R* = 0.3422, *p* < 0.05; fungi: ANOSIM *R* = 0.277, *p* < 0.05). Principal coordinate analysis based on Bray–Curtis dissimilarities clustered the prokaryotic and fungal communities according to sampling depth of the different humus depths, with the most distinct differences between the upper humus depths (0–10 and 10–20) and the deeper ones (Figure 4A,B). The clusters from the deeper depths overlapped slightly for prokaryotes and more strongly in the case of fungal communities (Figure 4A,B). For prokaryotes and fungi, a greater variability within groups (larger distance among the communities of one humus layer) was observed in the middle humus depths (10–20, 20–30 cm) compared to the uppermost and deepest depths, which clustered closer and were more homogenous (Figure 4A,B).

In total, 209 prokaryotic and 132 fungal biomarker taxa were identified in the five humus depths by LEfSe; by removing taxa with an LDA score lower than 3, 91 prokaryotic taxa and 24 fungal taxa remained as biomarkers for one specific humus depth. In general, more prokaryotic than fungal biomarkers were discovered in the different humus depths, and most of the biomarker taxa originated from either the uppermost (0–10 cm) or the deepest humus depth (40–50 cm). For the top humus depth (0–10 cm), the prokaryotic classes Gammaproteobacteria, Bacteroidia, Planctomycetes, Polyangia, and Saccharimonadia, and the fungal class Tremellomycetes were found to be biomarkers (Figure A7; Table A6). The prokaryotic classes Methylomirabilia, Gemmatimonadetes, Nitrososphaeria, RCP2‐54, Dadabacteriia, Nitrospiria were discovered as biomarkers for the deepest depth (40–50 cm), whereas no fungal biomarker was found at all for this depth (Figure A7; Table A6). No biomarker classes were detected within the three mid‐depths (10–20, 20–30, 30–40 cm).

## DISCUSSION

### Physical and chemical properties differentiate humus depths into different microbial habitats

As the deposition of plant litter on the top humus depth provides soil microorganisms with fresh OM (Liebmann et al., 2020), the Tangel humus top depth, with higher OM levels, should offer decomposing microorganisms better growth conditions than the deeper humus depths. Only a small part of litter‐derived OM finds its way to the deeper soil depths (via bioturbation), possibly causing decreased carbon turnover rates in the deeper depths (Liebmann et al., 2020). However, microbial degradation varies according to the water content, plant tissue type, and chemical composition and therefore determines whether OM is readily decomposed or stabilized within the humus formation of soil (Crow et al., 2009; Kögel‐Knabner, 2002). The C/N ratio is generally accepted as a measure of OM degradability (Blum, 2020), however, this ratio hardly changed along the sampled Tangel depth gradient. Consequently, the C/N ratio does not seem to be a good proxy for degradability in our defined depths of a Tangel humus. However, we found a higher, thus unfavourable TDC/TDN ratio within the deepest humus depth compared with the top layer. Besides C and N, a sufficient P supply is essential for microbial carbon turnover activity (Soong et al., 2020). We found that the P content was extremely low within the top depth of the Tangel humus (46.63 μg P g^−1^ DW) compared to forest soils on calcareous bedrock across North Tyrol (3.7 mg P g^−1^ DW) (Hofmann et al., 2016); it even further decreased in the deeper humus depths and this matches the findings of Wang et al. (2019) in a Bavarian Tangel humus profile. An inadequate P supply generally impedes microbial abundance and activity in soils (Cleveland & Liptzin, 2007), therefore, carbon turnover within the whole Tangel humus, and especially in the deeper humus depths, might be negatively affected. Nevertheless, P can also be unavailable for microorganisms due to the high phosphate sorption of humus layers (Giesler et al., 2005). Furthermore, the extremely low contents of readily available P in the deepest depth can be explained by the formation of insoluble calcium phosphates, favoured by the calcareous bedrock (even if small calcareous debris was also present throughout the whole profile) and the neutral pH in the deepest depths (Illmer & Schinner, 1995; Shen et al., 2011). So, a lower nutrient content in the deepest depths might have hampered microbial activity in these depths. Furthermore, the activity of microbial exoenzymes is often characterized by distinct pH optima (Leifeld et al., 2013; Neina, 2019). Most hydrolytic enzymes involved in OM degradation have distinct optima in the slightly acidic pH range (Turner, 2010). Thus, a lower soil pH in top humus layers might lead to higher availability of nutrients, higher activities of soil enzymes, and hence, to an increased carbon turnover in this depth. Besides pH, soil moisture is another well‐established driving factor of carbon turnover in soils (Abera et al., 2012), and we determined a lower (not significant) moisture content (increased DW) in the deeper humus depth, but the water content was still high (sufficient), whereas soil moisture may have not considerably influenced microbial activity.

### Abundance, activity, and diversity of the microbial communities in Tangel humus

A higher fungal abundance in the top depth compared with the deepest layer was most likely because of a higher input of fresh OM (consisting mainly of non‐readily‐biodegradable litter in the upper humus depths, providing good conditions for aerobic fungal growth) (van der Wal et al., 2013). The increase of total archaea abundance towards the deepest humus depth is in agreement with a previous study (Eilers et al., 2012) and might be because archaea prefer deeper depths with reduced oxygen supply and low OM availability (Malik et al., 2016). In contrast to archaea, bacteria play an important role in carbon decomposition (Domeignoz‐Horta et al., 2021) and they can use a wide range of aerobic and anaerobic pathways to process heterogeneous organic matter (Nunan et al., 2020). This catabolic versatility allows similar abundances in all humus depths despite different humus properties along the sampled Tangel humus. These results are confirmed by the behaviour of various ratios. The bacteria/fungi and archaea/fungi ratios increased significantly with depth, whereas the bacteria/archaea ratio decreased (Table 1), pointing to a more oxygen‐dependent fungal metabolism and a lower oxygen demand for archaea. Regarding microbial activity, we found that basal respiration (BR), a measure of aerobic carbon turnover activity, was significantly decreased in the deepest humus layer. The low activities of cellulolytic microorganisms in the deepest humus depth indicate that the decomposing microorganisms might face inadequate conditions (e.g., poor oxygen supply) for growth and decomposing activity, which corresponds to reduced fungal abundance. Microbial respiration calculated in relation to total carbon (Table 1; Figure A3D) (also true in relation to dissolved carbon) and cellulase activity per total or dissolved carbon decreased significantly with increasing depth, pointing to not only reduced absolute but also relative C turnover rates. Notably, in our sampled Tangel humus area, beech trees were the predominant forest species (Figure A9). The litter of beech trees exhibits high C/N ratios, hampering litter degradation (Berger et al., 2015). Similar to cellulase activity, the microbial biomass of metabolically active aerobic microorganisms (Cmic) was significantly lower in the deepest humus depth compared to the uppermost depth, again suggesting a weak nutrient and oxygen supply and indicating that the quality of the available OM does not meet the demands of the present microbial community (Bottino et al., 2016). Similarly, Cheng et al. (2013) reported that Cmic was positively correlated with the nutrient content (N, P). The decrease in microbial activity with increasing depth is not only evident in absolute measures but extends to the relative activities when normalised against bacterial or fungal abundances. Again, there was a significant decrease in the corresponding ratios (e.g., microbial respiration per microbial biomass, Table 1; Figure A3E), cellulase activity, and microbial respiration per bacterial or fungal abundance) as the depth increased. These findings are in line with our hypotheses that abiotic properties characterize our sampled depths specifically and microbial biomass and activity depend on humus depth. Apparently, not only the microbial activity was reduced but the metabolism seemed also to be less efficient, which resulted in a lower C turnover per microbial unit. Thus, our results indicate that carbon turnover in the deepest humus depth, with low fungal and high archaeal abundances, a specific microbial community, and lower microbial activity, was reduced compared to the top humus depths.

A higher microbial alpha diversity in soils can lead to a better performance in ecosystem functioning as more microbes are performing various functions (Wagg et al., 2019). Based on a previous study, a community with a high species diversity can use a broader range of resources than a community with a lower diversity (Bell et al., 2005). Maron et al. (2018) pointed out that differences in microbial alpha diversity may have an impact on microbial activity in soils. We could show that CA and BR corresponded not only to Cmic but also to microbial alpha diversity, with significantly higher levels in the top humus depth compared with the deepest humus depth. Apart from microbial alpha diversity per se, the top and deepest humus depths also differed significantly in their microbial community composition. We proved that the differences in the prokaryotic and fungal community composition in different humus depths were significantly associated with pH and the contents of OM, TDC, and TDN of the humus (Table A6). Similar results have been reported for other humus forms (e.g., Mediterranean Moder humus) (Andreetta et al., 2012). However, it remains unclear whether humus properties impact microbial communities or if they shape humus properties in the different humus depths.

### Microbial biomarkers emphasize differences between the top and deepest humus depths

We found that only the uppermost and deepest humus depths contained prokaryotic but also fungal biomarkers (at class level), demonstrating that the microbial community in the Tangel humus differed particularly between a depth of 0 and 40 cm. We used the known habitat requirements of the identified biomarker taxa to make predictions about the respective environmental conditions within the humus depths and to understand the significant differences in the microbial community among different humus depths.

Significantly higher relative abundances in the top humus depth compared with the deepest depth were indicated by random forest analysis, particularly for the prokaryotic classes Gammaproteobacteria, Planctomycetes, Bacteroidia, Polyangia, and Saccharimonadia, and confirmed by LefSe analysis. The relative abundances of these classes were positively correlated with the OM and TDN contents and negatively with pH (except for Gammaproteobacteria). This leads us to infer that the higher relative abundance of these classes in the top humus depth was possibly due to the higher nutrient content and lower pH conditions in this depth. Gammaproteobacteria are fast‐growing bacteria and are stimulated in soils with high amounts of readily available nutrients (Fierer et al., 2007; Pascault et al., 2013). Planctomycetes use various forms of carbon (Wiegand et al., 2018) and, similar to Bacteroidia (phylum Bacteroidetes), prefer environments with high nutrient levels (Fierer et al., 2007; Vitorino & Lage, 2022). Bacteroidia could be additionally promoted by a higher abundance of fungi in the top depths as they especially use fungal‐derived carbon (Clocchiatti et al., 2021). Polyangia belong to the phylum Myxococcota, whose representatives prey upon other microorganisms (Murphy et al., 2021). Therefore, their increased relative abundance might be due to a higher active microbial biomass on which they feed. Knowledge about the ecological preferences of Saccharimonadia (phylum Patescibacteria) is scarce. However, there is evidence in the literature that representatives of Saccharimonadia may live a symbiotic lifestyle due to their lack of essential biosynthetic capabilities (Lemos et al., 2019). Therefore, the significantly higher relative abundance in the top humus depth could be explained, apart from the higher nutrient content and lower pH, by the presence and/or higher abundance of microorganisms on whose metabolism they rely.

We proved a significantly higher abundance of all fungi in the top humus depth and significantly higher relative abundances, among others, particularly of the classes Orbiliomycetes and Tremellomycetes, of which the latter is a biomarker for the top humus depth. Tremellomycetes belong to the phylum Basidiomycota, contain fungi with various life forms and feeding strategies, and represent important players in wood rot activities and the mineralization of carbon compounds (Mašínová et al., 2018; Millanes et al., 2011). Although pH is an important factor influencing fungal growth in soil (Rousk et al., 2009), no significant correlation between pH and the abundance of Tremellomycetes could be established. In contrast, the relative abundance of Tremellomycetes correlated with the contents of TDN and OM, indicating that this class prefers higher nutrient levels, which is in agreement with the findings of a previous study (Köberl et al., 2020).

In the deepest humus layer, the relative abundances of Methylomirabilia, Gemmatimonadetes, Nitrososphaeria, Nitrospiria, Dadabacteriia, and RCP2‐54 were significantly increased, and these classes were even identified as biomarkers for the deepest humus depth. Their abundances were negatively correlated with the OM and TDN levels, indicating that they prefer or at least can cope with low nutrient contents. Indeed, some of the biomarker classes are autotrophic and do not rely on organic carbon. For example, the class Methylomirabilia consists of denitrifying methanotrophs that use methane as sole carbon and energy source (Rasigraf et al., 2014), whereas the class Nitrososphaeria (phylum Thaumarchaeota) includes ammonia‐oxidizing archaea that are adapted to oligotrophic environments (Saghaï et al., 2021; Schleper et al., 2005). The class Nitrospiria (phylum Nitrospirota) consists of diverse nitrite‐oxidizing bacteria that perform autotrophic carbon fixation (Lücker et al., 2010). Apart from their independence on carbon, their adaption to low oxygen levels is another characteristic that these classes (Methylomirabilia, Nitrososphaeria, Nitrospirota) share (Ettwig et al., 2010; Kraft et al., 2022; Lücker et al., 2010; Rasigraf et al., 2014). We therefore suggest that despite of the oligotrophic conditions, the low oxygen concentrations could have caused the significantly higher abundances of those classes in the deepest depth. The other biomarker classes that represent the deepest humus depth consist of the oligotrophic class Dadabacteriia, primarily using peptidoglycan of microbial necromass (Graham & Tully, 2021), and the class Gemmatimonadetes, a widespread class of generalists that prefer neutral a pH (DeBruyn et al., 2011). The habitat preferences of these two classes point to oligotrophic and neutral pH conditions in the deepest depth. Beyond the prokaryotic biomarker classes that prefer neutral pH, we could not detect any fungal biomarker for the deepest humus depth, indicating that the fungi avoid anaerobic, nutrient‐poor habitats. Overall, the environmental demands of the prokaryotic and fungal biomarker classes established are in line with the measurements of the physico‐chemical humus properties in this study.

## CONCLUSIONS

Our results show significant differences along a depth gradient of Tangel humus. This was a result of the differences in pH, nutrient availability, microbial abundance, activity, and community structure, thereby strongly affecting the carbon turnover of the Tangel humus and leading to the thick organic layers. The top humus depth exhibited higher nutrient levels (N, P), a lower soil pH, and higher microbial activity and alpha diversity compared with the deepest humus depth. Partial correlation analysis revealed that the OM, pH, P, and N levels were, among all investigated physical and chemical properties, the most important factors accounting for the differences among the Tangel humus depths. Prokaryotic and fungal community composition differed significantly between the top and the deepest humus depths, but it remains unclear whether the microbial communities are influenced only by the environmental conditions or whether they contribute to the altered humus properties. We discovered that fungal abundances (absolute and relative ones) highly contributed to the differences among the depths, and thus we assume that fungi play a key role in driving Tangel carbon turnover processes. In particular, fungi of the class Tremellomycetes, which occurred mainly in the upper depths, could have a major influence on the Tangel humus genesis. A just‐started investigation will therefore focus on the fungal carbon use potential, determined through the application of stable isotope investigations at the DNA level (DNA‐SIP). This will help to understand specific fungal groups involved in the degradation of soil organic matter as well as carbon use and transformation in Tangel systems, which is of particular interest in the context of climate change.

## AUTHOR CONTRIBUTIONS

**Theresa Rzehak:** Formal analysis; investigation; writing – original draft; writing – review and editing. **Nadine Praeg:** Conceptualization; formal analysis; investigation; writing – original draft; writing – review and editing. **Harald Zink:** Resources. **Alois Simon:** Resources. **Clemens Geitner:** Conceptualization; project administration; resources. **Paul Illmer:** Conceptualization; funding acquisition; project administration; supervision; writing – review and editing.

## CONFLICT OF INTEREST STATEMENT

The authors declare no conflicts of interest.

## Supporting information


**Data S1.** Supporting information.

## Data Availability

Raw sequencing data have been deposited in the NCBI Sequence Read Archive (SRA) and are accessible under the BioProject ID: PRJNA983232.
