# Supplementary material for: Microbial perspective of inhibited carbon turnover in Tangel humus of the Northern Limestone Alps
Source: Environ Microbiol Rep. 2023 Dec 7;16(1):e13215. doi: 10.1111/1758-2229.13215 (PMC10866079; doi:10.1111/1758-2229.13215)
Supplement: Supplementary file 1 — Data S1. Supporting information. [file EMI4-16-e13215-s001.docx]

***Environmental Microbiology Reports***

Appendix

**Microbial perspective of inhibited carbon turnover in Tangel humus of the Northern Limestone Alps**

Theresa Rzehak, Nadine Praeg, Harald Zink, Alois Simon, Clemens Geitner, Paul Illmer

**Selection of read count normalization method**

We applied four different normalization methods and compared their outcomes. We used the methods CLR (centered log ratios), CSS (cumulative sum scaling), rarefaction and TSS (total sum scaling). TSS was performed using the *hilldiv* package (Alberdi and Gilbert, 2019), resulting in a normalized OTU table consisting of proportions by dividing each OTU count by the total count of the library. To calculate CLR, zeros within the libraries were eliminated using the *zCompositions* package (Palarea-Albaladejo and Martín-Fernández, 2015), and subsequently, the normalized ratios were calculated by dividing each OTU count with the geometric mean of the library, applying the *phyloseq* package (McMurdie and Holmes, 2013). Cumulative sum scaling was performed using the *metagenomeSeq* package (Paulson et al., 2013). Feature counts were divided by the sum of counts up to a certain percentile, which was calculated using an associated *metagenomeSeq* function. Data were rarefied to even depth using the *phyloseq* package (McMurdie and Holmes, 2013) (prokaryotes: 30,238 reads per sample; fungi: 21,517 reads per sample). With every methods’ normalized output, a principal coordinate analysis (PCoA) was plotted using Bray-Curtis dissimilarities (CSS, rarefaction, TSS) or Euclidian dissimilarities (CLR) (Fig. A1 A, B). Furthermore, a PERMANOVA was performed with beta diversity estimates to test if the impact of sampling depth can explain variations of the community composition similarly for every normalization method’s output.

Indeed, outputs of all normalization methods showed a clustering according to sampling depth (Fig. A1 A, B, PERMANOVA p < 0.05), hence, produced equal results. We decided to use rarefaction as the normalization method of choice, because is it effectively accounts for different library sizes of the samples (Weiss et al. 2017).

**References**

**Gloor, G. B.; Macklaim, J. M.; Pawlowsky-Glahn, V.; Egozcue, J. J. (2017): Microbiome Datasets Are Compositional: And This Is Not Optional. In Front. Microbiol. 8, p. 2224. DOI: 10.3389/fmicb.2017.02224.**

**Alberdi, A., Gilbert, M.T.P., 2019. hilldiv: an R package for the integral analysis of diversity based on Hill numbers.**

**Palarea-Albaladejo, J., Martín-Fernández, J.A., 2015. zCompositions — R package for multivariate imputation of left-censored data under a compositional approach. Chemometrics and Intelligent Laboratory Systems 143, 85–96.**

**McMurdie, P.J., Holmes, S., 2013. phyloseq: An R Package for Reproducible Interactive Analysis and Graphics of Microbiome Census Data. PLOS ONE 8 (4), e61217.**

**Paulson, J.N., Stine, O.C., Bravo, H.C., Pop, M., 2013. Differential abundance analysis for microbial marker-gene surveys. Nat Methods 10 (12), 1200–1202.**

**Weiss, S; Xu, Z. Z.; Peddada, S.; Amir, A.; Bittinger, K.; Gonzalez, A. et al. (2017): Normalization and microbial differential abundance strategies depend upon data characteristics. In Microbiome 5 (1), p. 27. DOI: 10.1186/s40168-017-0237-y.**

Appendix figures: (Fig. A1 – Fig. A9)

**Fig. A1:** PCoA based on differently normalized data (normalization methods: CSS, rarefaction, CLR, TSS) for **A**: prokaryotic and **B**: fungal communities. Colors represent different humus depths [cm] and point shapes represent replicates. Eigenvalues explaining the variance of both dimensions are given in brackets. Results of PERMANOVA analysis among different humus depths are given as *p*-values in the respective plots.

**Fig.** **A2**: Humus chemical properties, including dry weight (A), pH (B), electrical conductivity (C), organic matter (D), total carbon (E), total nitrogen (F), phosphorus content (G) total dissolved nitrogen content (H), total dissolved carbon content (I), total dissolved organic carbon content (J), total carbon/total nitrogen ratio (K) and total dissolved carbon/total dissolved nitrogen ratio (L) in the Tangel humus depths. Shown are boxplots with medians (line), interquartile ranges of 50% (boxes) and minimum to maximum ranges (whiskers) (n = 3). Significant differences among humus depth [cm] are indicated by different letters.

**Fig.** **A3**: Microbial activity (cellulose activity, A and basal respiration, B), microbial biomass (microbial carbon content, C), the ratio of basal respiration to total carbon content (BR/TC, D) and basal respiration per unit of microbial biomass (metabolic quotient MQ, E) in the Tangel humus depths. Shown are boxplots with medians (line), interquartile ranges of 50% (boxes) and minimum to maximum ranges (whiskers) (n = 3). Significant differences among humus depth [cm] are indicated by different letters.

**Fig. A4:** Total abundance of archaea (**A**), bacteria (**B**) and fungi (**C**) and the respective ratios (**D**) in Tangel humus layers. Data were log-transformed. Shown are boxplots with medians (line), interquartile ranges of 50% (boxes) and minimum to maximum ranges (whiskers) (n = 3). Significant differences among humus depth [cm] are indicated by different letters.

fungi

prokaryotes

**Fig. A5:** Mean relative abundances (n=3) of the 10 most abundant members of the prokaryotic and fungal communities at class level in five Tangel humus depths [cm]. **A**: Prokaryotes, **B**: Fungi;

fungi

prokaryotes

**Fig. A6:** Mean relative abundances (n=3) of 50 most abundant prokaryotic (**A**) and fungal (**B**) families among five humus depths [cm]. Further prokaryotic families added to the bar “others”, mainly belong to the phyla Proteobacteria, Actinobacteriota, Chloroflexi, Acidobacteriota, Myxococcota. Further fungal families added to the bar “others”, mainly belong to the phyla Ascomycota, Basidiomycota, Rozellomycota, Chytridiomycota and Mucoromycota.

**Fig. A7:** Prokaryotic (upper panels) and fungal (lower panel) biomarkers (identified via LefSe) for the respective humus depth in the Tangel. Shown are boxplots with medians (line), interquartile ranges of 50% (boxes) and minimum to maximum ranges (whiskers) (n=3).

**Fig. A8:** RDA (redundancy analysis) ordination plot of the relationship between environmental factors and microbial communities among different humus depths [cm]. OM = organic matter, DW = dry weight, pH, EC = electrical conductivity, TC = total carbon content, TDC = total dissolved carbon, DOC = dissolved organic carbon, TN = total nitrogen content, TDN = total dissolved nitrogen, P = phosphorus content, CA = cellulase activity, BR = basal respiration, Cmic = microbial biomass; **A**: Prokaryotes, **B**: Fungi.


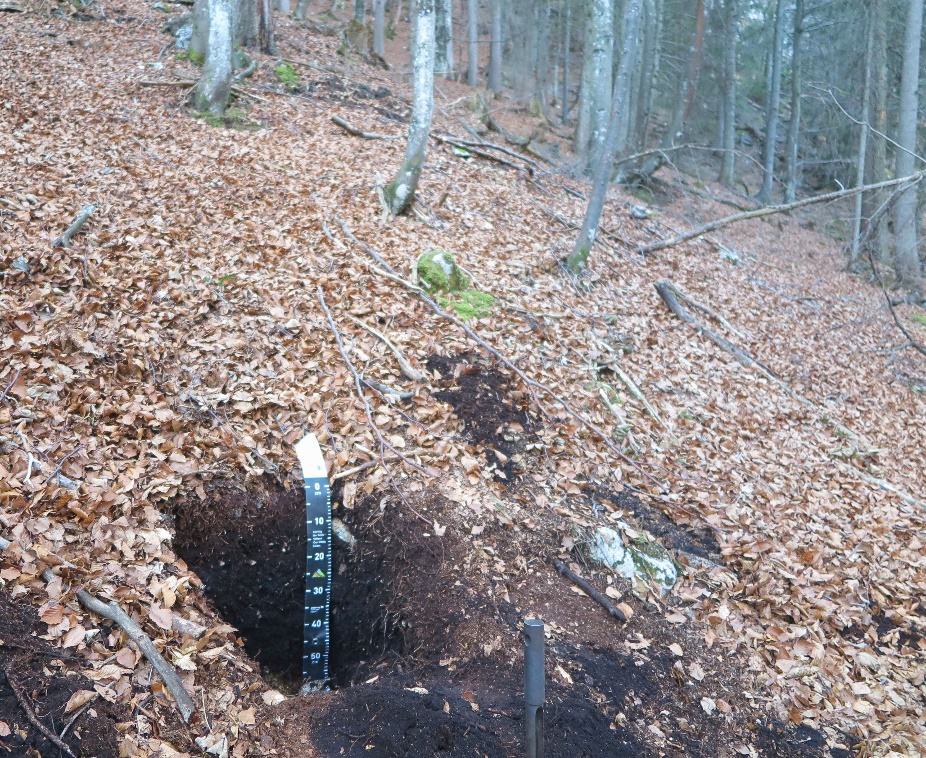


**Fig. A9:** This picture shows the forest site under investigation, located at a northwest exposed managed mixed-species forest (*Fagus sylvatica*, *Abies alba* and *Picea abies*) and the litter of the predominant vegetation.

Appendix tables (Tab. A1 – Tab. A8)

**Tab. A1:** Fractions of fine earth, mineral fragments, macro residues and roots among different humus depths [cm].

| **depth [cm]** |  | **fine earth** | **mineral fragments** | **macro residues** | **roots** | **total** |
| --- | --- | --- | --- | --- | --- | --- |
| 0-10 | [g] | 143.74 | 6.67 | 21.62 | 3.30 | 175.33 |
|  | [%] | 81.98 | 3.80 | 12.33 | 1.88 | 100.00 |
| 10-20 | [g] | 239.97 | 13.76 | 9.07 | 25.70 | 288.49 |
|  | [%] | 83.18 | 4.77 | 3.14 | 8.91 | 100.00 |
| 20-30 | [g] | 484.36 | 28.70 | 2.67 | 16.77 | 532.48 |
|  | [%] | 90.96 | 5.39 | 0.50 | 3.15 | 100.00 |
| 30-40 | [g] | 396.70 | 9.99 | 2.63 | 9.94 | 419.25 |
|  | [%] | 94.62 | 2.38 | 0.63 | 2.37 | 100.00 |
| 40-50 | [g] | 362.31 | 420.96 | 1.11 | 3.09 | 787.47 |
|  | [%] | 46.01 | 53.46 | 0.14 | 0.39 | 100.00 |

**Tab. A2:** Community composition of prokaryotes and fungi represented by relative abundances of top 10 most abundant classes within the Tangel humus;

| **prokaryotes** | | **fungi** | |
| --- | --- | --- | --- |
| **top 10 classes** | **relative abundance [%]** | **top 10 classes** | **relative abundance [%]** |
| Alphaproteobacteria | 22.17 | Agaricomycetes | 18.93 |
| Gammaproteobacteria | 9.06 | Sordariomycetes | 18.07 |
| Thermoleophilia | 7.28 | Leotiomycetes | 17.01 |
| Actinobacteria | 6.61 | Mortierellomycetes | 11.41 |
| Methylomirabilia | 5.83 | Saccharomycetes | 3.79 |
| Planctomycetes | 5.75 | Rozellomycotina_cls_Incertae_sedis | 3.38 |
| Vicinamibacteria | 5.27 | Tremellomycetes | 2.11 |
| Acidobacteriae | 5.00 | Microbotryomycetes | 2.09 |
| Verrucomicrobiae | 4.26 | Eurotiomycetes | 1.75 |
| Blastocatellia | 3.82 | Agaricostilbomycetes | 1.18 |

**Tab. A3:** Mean relative abundances [%] of ten most abundant prokaryotic and fungal classes respectively among different humus depths [cm]. The intensity of the grey tones indicates the extend of the relative abundance. *p*-values are derived from Kruskal-Wallis test among humus depths and values marked with an asterisk denote significant differences in relative abundance among depths (*p* < 0.05).

|  |  | **depth [cm]** | | | | |  |
| --- | --- | --- | --- | --- | --- | --- | --- |
|  | **class** | **0-10** | **10-20** | **20-30** | **30-40** | **40-50** | ***p*-value** |
| **prokaryotes** | Acidobacteriae | 6.65 | 7.65 | 6.53 | 3.19 | 0.97 | 0.187 |
|  | Actinobacteria | 7.76 | 8.89 | 8.28 | 5.63 | 2.50 | 0.097 |
|  | Alphaproteobacteria | 23.52 | 24.29 | 23.10 | 21.61 | 18.34 | 0.107 |
|  | Blastocatellia | 2.03 | 3.46 | 3.84 | 4.43 | 5.36 | 0.371 |
|  | Gammaproteobacteria | 15.37 | 9.41 | 6.61 | 6.69 | 7.24 | **0.022*** |
|  | Methylomirabilia | 1.45 | 3.83 | 6.01 | 7.95 | 9.91 | **0.017*** |
|  | others | 21.20 | 19.26 | 23.70 | 27.27 | 33.33 | 0.176 |
|  | Planctomycetes | 6.76 | 6.19 | 6.07 | 5.16 | 4.57 | 0.029 |
|  | Thermoleophilia | 6.57 | 7.01 | 6.93 | 7.91 | 7.97 | 0.393 |
|  | Verrucomicrobiae | 5.47 | 6.36 | 4.69 | 3.48 | 1.27 | 0.088 |
|  | Vicinamibacteria | 3.23 | 3.65 | 4.24 | 6.70 | 8.53 | 0.116 |
| **fungi** | Agaricomycetes | 24.36 | 26.89 | 19.04 | 13.00 | 11.39 | 0.448 |
|  | Agaricostilbomycetes | 0.68 | 0.78 | 0.98 | 1.84 | 1.63 | 0.669 |
|  | Eurotiomycetes | 2.38 | 2.45 | 1.35 | 1.45 | 1.11 | 0.085 |
|  | Leotiomycetes | 14.03 | 13.27 | 22.72 | 18.00 | 17.02 | 0.705 |
|  | Microbotryomycetes | 0.76 | 2.22 | 3.44 | 3.05 | 0.98 | 0.598 |
|  | Mortierellomycetes | 12.88 | 11.78 | 7.58 | 10.44 | 14.37 | 0.240 |
|  | others | 25.15 | 22.59 | 15.96 | 21.68 | 16.00 | 0.803 |
|  | Rozellomycotina_cls_Incertae_sedis | 2.85 | 2.50 | 2.78 | 3.99 | 4.80 | 0.779 |
|  | Saccharomycetes | 1.11 | 6.53 | 5.17 | 4.95 | 1.19 | 0.669 |
|  | Sordariomycetes | 9.66 | 8.32 | 20.15 | 21.04 | 31.19 | 0.212 |
|  | Tremellomycetes | 6.16 | 2.68 | 0.83 | 0.55 | 0.32 | **0.034*** |

**Tab. A4:** Diversity measures (Richness, Shannon, Simpson) among five humus depths [cm] for prokaryotic and fungal communities. Asterisks behind p-values (ANOVA) mark significant differences (*** p* < 0.01, ** p* < 0.05) of diversity measures among different depths [cm].

|  | **prokaryotes** | | | **fungi** | | |
| --- | --- | --- | --- | --- | --- | --- |
| **depth [cm]** | Richness | Shannon | Simpson | Richness | Shannon | Simpson |
| 0-10 | 2456 | 475 | 153 | 742 | 62 | 19 |
| 10-20 | 2557 | 390 | 125 | 607 | 55 | 21 |
| 20-30 | 2152 | 313 | 107 | 507 | 22 | 9 |
| 30-40 | 2103 | 333 | 113 | 478 | 26 | 11 |
| 40-50 | 1887 | 323 | 116 | 476 | 28 | 9 |
| ***p*-value** | **0.036*** | **0.040*** | 0.239 | **0.004**** | 0.066 | **0.039*** |

**Tab. A5:** Pearson correlations between environmental variables and microbial communities (Bray-Curtis dissimilarities) using a Mantel test. Numbers represent the r values (correlation) and the asterisks the significances (*** p < 0.001, ** p < 0.01)

|  | **prokaryotes** | **fungi** |
| --- | --- | --- |
| OM | 0.864 *** | 0.681 *** |
| pH | 0.846 *** | 0.634 *** |
| TDC | 0.362 ** | 0.293 ** |
| TDN | 0.431 ** | 0.405 ** |

**Tab. A6:** LDA scores and *p* values given for microbial biomarker classes that were identified for different humus depths [cm] by the LEfSe method.

|  | **Class** | **depth [cm]** | ***p* value** | **LDA score** |
| --- | --- | --- | --- | --- |
| **prokaryotes** | Gammaproteobacteria | 0-10 | 0.022 | 4.64 |
|  | Bacteroidia | 0-10 | 0.011 | 4.08 |
|  | Planctomycetes | 0-10 | 0.029 | 3.92 |
|  | Polyangia | 0-10 | 0.022 | 3.77 |
|  | Saccharimonadia | 0-10 | 0.032 | 3.31 |
|  | Methylomirabilia | 40-50 | 0.017 | 4.51 |
|  | Gemmatimonadetes | 40-50 | 0.023 | 4.13 |
|  | Nitrososphaeria | 40-50 | 0.023 | 3.58 |
|  | RCP2-54_cl | 40-50 | 0.029 | 3.55 |
|  | Dadabacteriia | 40-50 | 0.011 | 3.44 |
|  | Nitrospiria | 40-50 | 0.033 | 3.36 |
| **fungi** | Tremellomycetes | 0-10 | 0.034 | 4.08 |

**Tag. A7:** Microbial classes exhibiting significantly different relative abundances (p < 0.05) among humus depths were identified by random forest analysis. Classes are ranked according to their MeanDecreaseGini value, indicating the importance of each microbial class.

|  | **Class** | ***p* value** | **MeanDecreaseGini** |
| --- | --- | --- | --- |
| **prokaryotes** | Dadabacteriia | 0.011 | 1.109 |
|  | Bacteroidia | 0.011 | 1.075 |
|  | Planctomycetes | 0.029 | 0.861 |
|  | Gammaproteobacteria | 0.022 | 0.769 |
|  | RCP2-54_cl | 0.029 | 0.753 |
|  | Gemmatimonadetes | 0.023 | 0.704 |
|  | Oligoflexia | 0.022 | 0.689 |
|  | Methylomirabilia | 0.017 | 0.685 |
|  | Nitrososphaeria | 0.023 | 0.640 |
|  | Polyangia | 0.022 | 0.622 |
|  | Armatimonadia | 0.013 | 0.565 |
|  | Fimbriimonadia | 0.031 | 0.478 |
|  | Nitrospiria | 0.033 | 0.432 |
|  | Lineage_IIa | 0.047 | 0.424 |
|  | Saccharimonadia | 0.032 | 0.417 |
|  | Parcubacteria | 0.049 | 0.373 |
|  | GAL15_cl | 0.043 | 0.339 |
|  | vadinHA49 | 0.021 | 0.265 |
| **fungi** | Orbiliomycetes | 0.013 | 1.821 |
|  | Endogonomycetes | 0.030 | 1.534 |
|  | Tremellomycetes | 0.034 | 1.524 |
|  | GS37 | 0.022 | 1.376 |
|  | Rhizophydiomycetes | 0.042 | 1.047 |
|  | Sanchytriomycetes | 0.008 | 1.027 |
|  | Umbelopsidomycetes | 0.043 | 0.989 |
|  | Lobulomycetes | 0.040 | 0.694 |
|  | Mucoromycetes | 0.031 | 0.621 |
|  | GS19 | 0.018 | 0.419 |
